# Is Interoception Deficit Linking Alexithymia and Eating Spectrum Symptoms? Study on a Non-Clinical Sample of Young Adults

**DOI:** 10.3390/healthcare12050586

**Published:** 2024-03-04

**Authors:** Mario Miniati, Maria Lippi, Laura Palagini, Ciro Conversano, Graziella Orrù, Angelo Gemignani, Giulio Perugi

**Affiliations:** 1 Psychiatric Clinic, Department of Clinical and Experimental Medicine, University of Pisa, 56126 Pisa, Italy; lpalagini@tiscali.it (L.P.); giulio.perugi@med.unipi.it (G.P.); 2 Department of Surgical, Medical and Molecular Pathology and of Critical Care Medicine, University of Pisa, 56126 Pisa, Italy; m.lippi12@studenti.it (M.L.); ciro.conversano@med.unipi.it (C.C.); graziella.orru@unipi.it (G.O.); angelo.gemignani@unipi.it (A.G.)

**Keywords:** alexithymia, eating disorders spectrum, anorexia nervosa, bulimia nervosa, interoception

## Abstract

We investigated if interoceptive deficits could be the link between alexithymic traits and eating spectrum manifestations in a non-clinical sample. One-hundred sixty-one young adults (mean age: 23.2 ± 2.4 years) were evaluated with the Toronto Alexithymia Scale-20 (TAS-20), the Interoceptive Accuracy Scale (IAS), the Interoceptive Confusion Questionnaire (ICQ), and the Eating Attitudes Test-26 (EAT-26). Questionnaires were administered with an online procedure (Microsoft Form, Office 365 A1, Pisa, Italy) (Study Protocol #0012005/2023). We compared ICQ, IAS, and TAS-20 scores in subjects who met the threshold for a potential eating spectrum disorder according to EAT-26 scores ≥ 20 (*n* = 27) vs. subjects who scored <20 (*n* = 134), with an ANCOVA corrected for ‘age’ and ‘gender’. Subjects with EAT-26 ≥ 20, scored significantly higher at ICQ (54.4 ± 13.2 vs. 50.2 ± 6.8; *p* = 0.011), TAS-20 ‘Total Score’ (60.8 ± 11.9 vs. 58.1 ± 9.2; *p* = 0.006), and TAS-20 ‘Identifying Feelings’ (21.5 ± 7.6 vs. 17.3 ± 5.8; *p* = 0.0001). A binary logistic regression analysis, with EAT-26 scores < 20 vs. ≥20 as the dependent variable, and ICQ, IAS, TAS-20 total scores and dimensions, age, and gender (categorical) as covariates, showed that the only variable predicting eating spectrum symptomatology was ‘ICQ Total Score’ (OR = 1.075, 95% CI: 1.016–1.139; *p* = 0.013). Interoceptive confusion was the dimension linking the occurrence of alexithymic traits and eating spectrum manifestations.

## 1. Introduction

The eating disorders’ spectrum (EDs) includes several different clinical conditions, such as Anorexia Nervosa Restricting Subtype (AN-R), bingeing-purging subtype (AN-BP), Bulimia Nervosa (BN), and Binge Eating Disorder (BED) (DSM-5) [1]. EDs are heterogeneous, with long-term diagnostic instability and overlapped categorical boundaries, including atypical eating, weight-control behaviors, altered perception of body dimensions/shapes, and impulsiveness [2,3,4]. The prevalence of full-blown disorders is reaching 2.2% in Europe, 3.5% in Asia, and 4.6% in USA; sub-threshold or atypical forms are not included in these percentages [5,6,7]. Taken as a whole, EDs are associated with high mortality rates and physical/psychological comorbidities, namely depressive episodes (MDE), bipolar disorders (BP), obsessive-compulsive disorder (OCD), panic disorder (PD), post-traumatic spectrum disorders (PTSD), and autism spectrum disorders (ASD), all interfering with the response to available treatment options, especially in patients with AN [8,9,10,11,12,13]. Patients with AN and ASD seem to share a similar neuropsychological background, demonstrating impairment in emotion recognition, empathic abilities, and emotional introspection [14], leading to conditions characterized by intense ‘interpersonal discomfort’ [15] and chronic severe difficulties in social activities [16].

Recently, a role for ‘alexithymic traits’ has been hypothesized both for AN and ASD patients. Alexithymia has been characterized by difficulties in identifying and describing feelings (one’s own and those of others), and by an externally oriented cognitive style [17,18]. Emotion recognition difficulties or deficit in empathy and interoception, typical of both AN and ASD patients, have been interpreted as deriving from a pre-morbid ‘alexithymic dimension’ [19]. Subjects with alexithymic traits find it difficult to discriminate internal feelings from physical sensations and may show marked deficits in empathy, emotion recognition, and regulation [17].

Alexithymia has been associated with low levels of interoceptive accuracy and is considered to be an epiphenomenon of a pre-existing impaired interoception [19,20]. Interoception consists of three different components: accuracy, sensibility, and awareness [21]. Interoceptive accuracy is the ability to detect internal bodily states (such as counting one’s heartbeat) [21]; interoceptive sensibility is the ability to correctly evaluate one’s own internal bodily states [21]; interoceptive awareness is the ability to recognize, understand, and react to internal bodily states [21,22,23]. As far as we know, few studies have explored the potential relationships between EDs and alexithymia or between EDs and interoceptive deficits, both in clinical and non-clinical samples [24,25,26]. 

The main aim of our study was to explore, in a general population sample of young adults of all genders, the potential relationships between three dimensions, namely sub-threshold signs and symptoms of eating disorders spectrum, alexithymic traits, and altered interoception. Our hypothesis was threefold: (a) subjects from the general population might have different level of interoceptive abilities; (b) low interoceptive awareness and high interoceptive confusion might predispose a subject to difficulties in identifying/describing feelings, commonly considered as ‘alexithymic traits’; (c) we then hypothesized that subjects with interoceptive confusion, low levels of interoceptive awareness, and high levels of alexithymic traits might be more prone to eating spectrum signs and symptoms than subjects without these characteristics. 

## 2. Materials and Methods

### 2.1. Participants and Procedures 

The study had a cross-sectional, observational, and non-profit design. A total of 161 young community volunteers were enrolled (starting in March 2023 and ending in April 2023) via an online procedure. A link to the self-administered questionnaire was publicized on the most common social networks. Interested subjects read a brief online description of the study, then they completed the informed consents (consent to participate and consent for the privacy issues). Participants could then have access to the questionnaires. To be included in the study, participants had to be aged between 18 and 30 years and be without any severe physical illness or current psychiatric disorder, including substance or alcohol use/abuse or suicidal ideation. However, no measures were taken to verify the actual satisfaction of such criteria. In that case, it cannot be excluded that there were subjects in the group examined who did not meet the stated inclusion criteria.

The Bioethics Committee of the University of Pisa approved the consents and the study (Prot. #0012005/2023). All study procedures were conducted in accordance with the Helsinki Declaration.

### 2.2. Questionnaires

Demographics including age, gender, relationship status, working activity, education, and area of residence (urban/suburban/rural) were collected. Four self-administered scales were utilized. These included a questionnaire considered the gold standard for the detection of alexithymic traits (Toronto Alexithymia Scale-20-TAS-20) [27], two questionnaires exploring interoceptive accuracy and confusion, namely the Interoceptive Accuracy Scale-IAS [28] and the Interoceptive Confusion Questionnaire (ICQ) [20], respectively, and one assessing dysfunctional eating behaviors and habits (Eating Attitudes Test-26 EAT-26) [29].

#### 2.2.1. Toronto Alexithymia Scale-20-TAS-20) [27]

TAS-20 is a 20-item questionnaire rated on a 5-point Likert scale ranging from ‘1′ (‘strongly disagreeing’) to ‘5′ (‘strongly agreeing’). The instrument explores three dimensions of alexithymia: (1) identification of one’s own emotional states or ‘difficulty identifying feelings’ (DIF); (2) the ability to verbally describe emotional states to others or ‘difficulty describing feelings’ (DDF); (3) and the so-called ‘inclination away from introspection and towards externally-orientated thinking’ (EOT). Total scores > 61 (range 20–100) indicate a relevant level of impairment. TAS-20 showed good internal consistency in the literature (Cronbach’s α = 0.81) [27], as confirmed in our study (Cronbach’s α = 0.73).

#### 2.2.2. Interoceptive Accuracy Scale (IAS) [21,28]

IAS aims to evaluate the interoceptive sensitivity, defined as the ability to understand internal sensations more or less accurately. The scale is made up of 21 items and measures how much the subjects believe they are able to accurately perceive specific bodily sensations. Subjects are asked to indicate, on a Likert scale from 1 = ‘strongly disagree’ to 5 = ‘strongly agree’, the ‘degree of affinity’ with what is expressed by each item. Higher scores, obtained through the sum of the scores given for each item, indicate a more accurate ability of subjective interoceptive sensitivity, while low scores indicate low accuracy. There are no reverse items. IAS demonstrated excellent internal consistency in our study (Cronbach’s α = 0.90).

#### 2.2.3. Interoceptive Confusion Questionnaire (ICQ) [20]

ICQ is a questionnaire made up of 20 items that explore the levels of interoceptive accuracy/inaccuracy of what is subjectively perceived regarding states such as hunger, muscle soreness, or levels of arousal. Each item is rated on a Likert scale from 1 to 5, with the total score given by each item. A higher score corresponds to a greater accuracy in the interpretation of interoceptive stimuli. In our study, ICQ showed acceptable internal consistency (Cronbach’s α = 0.60).

#### 2.2.4. Eating Attitudes Test-26 (EAT-26) [29]

EAT-26 is the gold standard test to measure symptoms and beliefs characteristics of eating disorders, even if does not allow the clinician to make a specific diagnosis. EAT-26 consists of 26 questions rated on a six-point scale, from 0 (‘Never’) to 3 (‘always’), and is considered an efficient screening tool in general population samples (total score ranging from 0 to 78). Subjects who score 20 or higher are candidates for having an eating disorders spectrum diagnosis. The Cronbach’s α of the questionnaire in our study has been rated as 0.74.

### 2.3. Statistical Analyses

We described quantitative variables by means and/or medians and standard deviations. Qualitative variables were expressed with frequencies and percentages. The Kolmoronov–Smirnov test was applied to evaluate whether the variables studied had a normal distribution. For the same purposes, means, medians, distribution, and kurtosis were also assessed. The ANOVA test was used to compare the values of variables with Gaussian distribution. In the case of non-Gaussian variables, the Kruskal–Wallis test was applied. Any differences or associations between nominal variables were evaluated with the Chi-square test or Fisher’s exact test, depending on the frequencies detected. Differences between values were analyzed with the *T*-Test for independent samples, when appropriate; for non-Gaussians variables, the Wilcoxon test was performed. An ANCOVA adjusted for the variables ‘age’ and ‘gender’ was applied. Correlation analyses were performed using both Spearman’s and Pearson’s tests, depending on the distribution of the variables considered. The predictive validity of both alexithymic traits and interoceptive dimensions for the severity of eating spectrum symptoms in the overall sample was assessed with a binary logistic regression analysis, with EAT-26 < 20 vs. ≥20 as the dependent variable. The level of statistical significance was associated with *p* < 0.05. Moreover, we performed a mediation model with the aim to test direct and mediated effects relating TAS, ICQ, IAS, and EAT-26 dimensions. Sample size was calculated with a ‘a priori’ power analysis. Analyses were carried out using IBM SPSS Statistics.

## 3. Results

### 3.1. Overview

Our sample consisted of 161 young adults (47 males, 29.2%, and 114 females, 70.8%). The mean age was 23.2 ± 2.4 years (range: 18–30). Mean age was significantly different in males and females (M: 23.7 ± 2.4 vs. F: 22.9 ± 2.3; *p* = 0.018). Sociodemographic information is summarized in Table 1. 

### 3.2. Scores of the Administered Scales

Scores of the administered scales in the overall sample and by gender are summarized in Table 2. Considering that mean age was significantly different in males and females, we performed an ANCOVA, using ‘age’ as a covariate, when comparing the two genders. Females scored significantly higher than males in a number of scales and sub-scales, namely: EAT-26 ‘Total Score’ (12.6 ± 11.1 vs. 11.2 ± 8.0; *p* = 0.003), EAT-26 ‘Dieting’ (6.1 ± 6.6 vs. 5.2 ± 5.7; *p* = 0.007), EAT-26 ‘Bulimia/Food Preoccupation’ (4.5 ± 2.7 vs. 4.2 ± 2.2; *p* = 0.19), EAT-26 ‘Oral Control’ (1.9 ± 3.0 vs. 1.6 ± 2.5; *p* = 0.042), TAS-20 ‘Total Score’ (59.2 ± 9.6 vs. 56.9 ± 9.8; *p* = 0.002), and TAS-20 ‘Identifying Feelings’ (18.6 ± 6.4 vs. 16.5 ± 5.9; *p* = 0.0001). No differences were found on ICQ and IAS scores, nor on the distribution of subjects who fulfilled the threshold for a potential eating disorder, according to EAT-26 score (≥20), or for alexithymia, according to TAS-20 score (>61).

#### 3.2.1. ICQ, IAS and TAS-20 in Subjects with EAT-26 Score < 20 vs. EAT-26 Score ≥ 20

We compared ICQ, IAS, and TAS-20 scores in subjects who fulfilled the threshold for a potential eating spectrum disorder, according to EAT-26 scores (≥20) (*n* = 27) vs. subjects who scored <20 (*n* = 134), performing an ANCOVA, corrected for ‘age’ and ‘gender’. Subjects with EAT-26 scores ≥ 20 scored significantly higher at ICQ (54.4 ± 13.2 vs. 50.2 ± 6.8; *p* = 0.011), TAS-20 ‘Total Score’ (60.8 ± 11.9 vs. 58.1 ± 9.2; *p* = 0.006), and TAS-20 ‘Identifying Feelings’ (21.5 ± 7.6 vs. 17.3 ± 5.8; *p* = 0.0001), as summarized in Table 3.

#### 3.2.2. ICQ, IAS, and EAT-26 Scores in Subjects with TAS-20 Score > 61 vs. TAS-20 Score ≤ 61

We compared interoception measures and eating spectrum dimensions in subjects who fulfilled the criterion for the alexithymia diagnosis (TAS-20 > 61) (*n* = 58) vs. subjects who scored below the TAS-20 threshold (TAS-20 ≤ 61) (*n* = 103). Subjects with alexithymia scored significantly higher on ICQ ‘Total Score’ (54.7 ± 8.4 vs. 48.7 ± 7.5; *p* = 0.0001), EAT-26 ‘Total score’ (13.6 ± 8.7 vs. 11.4 ± 11.0; *p* = 0.008), and EAT-26 ‘Dieting’ (6.9 ± 5.5 vs. 5.3 ± 6.6; *p* = 0.013), as summarized in Table 4.

### 3.3. Correlation Analyses

Correlations analyses were interpreted with the Pearson r or the Spearman rs coefficients, where appropriate, on self-report administered scales’ scores. 

TAS-20, ICQ, and IAS scores had a normal distribution in the overall sample; EAT-26 ‘Total Score’, ‘Dieting’, ‘Bulimia/Food Preoccupation’, and ‘Oral Control’ showed non-parametric distributions, according to the Kolmogorov–Smirnov Test. Correlations are summarized in Table 5a–c.

In the overall sample (*n* = 161) (Table 5a), ICQ ‘Total Score’ was positively correlated with TAS-20 ‘Total Score’ (r = 0.383; *p* = 0.001), and ‘Identifying Feelings’, and with EAT-26 ‘Total Score’ (r = 0.302; *p* < 0.01), ‘Dieting’ (r = 0.247; *p* < 0.01), and ‘Bulimia/Food Preoccupation’ (r = 0.232; *p* < 0.01). The IAS ‘Total Score’ was negatively correlated with TAS-20 ‘Total Score’ (r = −0.359; *p* < 0.01), ‘Identifying Feelings’ (r = −0.460; *p* < 0.01), ‘Describing Feelings’ (r = −0.230; *p* < 0.01), and with EAT-26 ‘Total Score’ (r = −0.170; *p* < 0.05). The TAS-20 ‘Total Score’ was positively correlated also with EAT-26 ‘Total Score’ (r = 0.269; *p* < 0.01), ‘Dieting’ (r = 0.226; *p* < 0.01), and ‘Bulimia/Food Preoccupation’ (r = 0.182; *p* < 0.05). The TAS-20 ‘Identifying Feelings’ was positively correlated with EAT-26 ‘Total Score’ (r = 0.360; *p* < 0.01), ‘Dieting’ (r = 0.301; *p* < 0.01), ‘Bulimia/Food Preoccupation’ (r = 0.264; *p* < 0.01), and ‘Oral Control’ (r = 0.195; *p* < 0.05). 

In males’ sample (*n* = 47) (Table 5b), ICQ Total Score was positively correlated with TAS-20 ‘Total Score’ (r = 0.297; *p* < 0.05), with TAS-20 ‘Identifying Feelings’ (r = 0.393; *p* < 0.01), with EAT-26 ‘Total Score’ (rs = 0.255; *p* < 0.01), and ‘Bulimia/Food Preoccupation’ (rs = 0.393; *p* < 0.01). IAS ‘Total Score’ was negatively correlated with TAS-20 ‘Total Score’ (r = −0.435; *p* < 0.01), with TAS-20 ‘Identifying Feelings’ (r = −0.333; *p* < 0.05), and with TAS-20 ‘Describing Feelings’ (r = −0.465; *p* < 0.01). The TAS-20 ‘Total Score’ was not significantly correlated with EAT-26 scores, whereas TAS-20 ‘Identifying Feelings’ was positively correlated with EAT-26 ‘Total Score’ (rs = 0.333; *p* < 0.05) and with EAT-26 ‘Bulimia/Food Preoccupation’ (rs = 0.301; *p* < 0.05). TAS-20 ‘Describing Feelings’ and ‘EOT’ were not correlated with EAT-26 scores. 

In females’ sample (*n* = 114) (Table 5c), ICQ ‘Total Score’ was positively correlated with TAS-20 ‘Total Score’ (r = 0.451; *p* < 0.01), with TAS-20 ‘Identifying Feelings’ (r = 0.503; *p* < 0.01), with TAS-20 ‘EOT’ (r = 0.189; *p* < 0.05), with EAT-26 ‘Total Score’ (rs = 0.255; *p* < 0.01), and with EAT-26 ‘Bulimia/Food Preoccupation’ (rs = 0.393; *p* < 0.01). IAS ‘Total Score’ was negatively correlated with TAS-20 ‘Total Score’ (r = −0.334; *p* < 0.01) and with TAS-20 ‘Identifying Feelings’ (r = −0.493; *p* < 0.01). Moreover, IAS ‘Total Score’ was negatively correlated with EAT-26 ‘Total Score’ (rs = −0.244; *p* < 0.01). The TAS-20 ‘Total Score’ was positively correlated with EAT-26 ‘Total Score’ (rs = 0.310; *p* < 0.01). TAS-20 ‘Identifying Feelings’ was positively correlated with EAT-26 ‘Total Score’ (rs = 0.373; *p* < 0.01) and with EAT-26 ‘Bulimia/Food Preoccupation’ (rs = 0.301; *p* < 0.05).

### 3.4. Binary Logistic Regression Analysis of Subjects with EAT-26 Total Score <20 vs. ≥20

A binary logistic regression analysis was performed with the aim to evaluate if alexithymic and/or interoceptive dimensions are able to predict the presence of a more severe eating spectrum symptomatology in our sample. The EAT-26 cut-off score < 20 vs. ≥20 was utilized as the dependent variable; age and gender (categorical), ICQ, IAS, and TAS-20 total scores were inserted as covariates. The ICQ Total Score was the only variable in our model that predicted for a more relevant eating spectrum symptomatology, even if with a low odds ratio (OR = 1.075, 95% CI: 1.016–1.139; *p* = 0.013), as summarized in Table 6.

### 3.5. Mediation Analyses

Mediation analyses were performed with the aim to check the potential processes underlying the relationships between the considered variables (namely, ICQ, IAS, TAS-20, and EAT-26 scores). All mediation pathways were explored. No statistically significant finding emerged.

## 4. Discussion

Interoception and its alterations have been extensively explored in eating spectrum disorders [23,24,30]. Furthermore, alexithymia and its correlations with interoceptive deficit/confusion have been investigated, especially in patients with AN [31,32,33,34,35]. As far as we know, few studies have examined both interoception alterations and alexithymic traits in non-clinical samples with the aim to evaluate potential proneness to eating spectrum symptoms [24,25,26]. Our study confirmed that eating spectrum signs and symptoms were more frequent among females than males in the general population, even if not reaching the threshold for a full-blown eating disorder. Alterations in dietary habits, bulimic traits, food preoccupation, and difficulties in balancing food control were the most relevant psychological dimensions, as already reported in previous observations [4,36,37]. Moreover, we found that females showed more relevant alexithymic traits than males (as detected by the TAS-20), especially in the domain of an inadequate identification of feelings. This finding is partially in line with the literature and could be explained with the more frequent occurrence of eating spectrum traits in females than in males: the ‘psychopathological core’ of alexithymia is characterized by a marked difficulty in emotions’ identification and elaboration and by a significant interference with self-regulation processes predisposing to dysfunctional eating behaviors, such as binge-eating [38,39,40] or emotional eating [41]. However, several studies pointed out that alexithymic traits might be more frequent among males than females as a consequence of the so-called ‘normative male alexithymia’ [42], characterized by a deficit in processing emotions. According to this hypothesis, alexithymia in males could be the result of emotions’ dissociation, repression or suppression, or the consequence of a deficit in identifying words for expression of vulnerability and attachment, always discouraged by ‘masculine norms’ [42].

Correlation analyses performed on our sample confirmed that there was a relationship between alteration of interoception, alexithymic traits, and eating spectrum dimensions. IAS scores were negatively correlated (the highest IAS scores were representative of a more accurate interoception) and ICQ scores were positively correlated (the highest ICQ scores were representative of a more severe interoceptive confusion) with EAT-26 and TAS-20 domains.

Moreover, when we performed an ANCOVA analysis, corrected for ‘age’ and ‘gender’, in order to compare subjects with EAT scores< vs. ≥20 (namely subjects with and without sub-threshold pathological eating traits and habits), we found that subjects with more severe eating traits (EAT scores ≥ 20) were characterized by interoceptive confusion (scored with the ICQ) and by alexithymic traits, with especially marked difficulties in identifying feelings. This finding is confirmed in our sample by the evidence of higher EAT-26 scores in subjects with more severe alexithymic traits (TAS-20 > 61), especially for the ‘dieting’ habits.

In order to detect which variable could predict for a more relevant eating spectrum symptomatology, we performed a binary logistic regression analysis, with EAT-26 scores < 20 vs. ≥20 as the dependent variable and ICQ, IAS, and TAS-20 total scores and dimensions, age, and gender (categorical) as the covariates. We found that the only variable that predicted more frequent eating spectrum signs and symptoms was the ICQ Total Score, but with a low odds ratio (OR = 1.075, 95% CI: 1.016–1.139; *p* = 0.013); this was perhaps due to the fact that the assessment was performed on a non-clinical sample, with sub-threshold eating manifestations.

This finding is in line with a study that considered a valid interoceptive capacity as a prerequisite for adequate emotions’ experiences [22], and with a second more recent study on three general populations’ samples (from Italy, the U.S., and Singapore) of subjects with scarce interoceptive skills (measured with ICQ) that showed more alexithymic traits when compared to subjects with good interoception [21]. The role of interoceptive confusion as predictor of eating signs and symptoms has been also found in a study conducted on 213 university students, in which dysfunctional processing of hunger and satiety were important risk factors for disordered eating [43]. Studies on clinical samples showed that EDs diagnoses might moderate the association between alexithymic traits and emotions’ dysregulation, impulse control difficulties, and access to emotion regulation strategies, with a greater impact on emotion dysregulation for patients with AN [31]. This finding has been confirmed by evidence that training in interoceptive skills, especially on sensitivity and awareness, might be able to reduce severity of eating spectrum symptoms [44]. However, for patients with AN, other variables have been traditionally considered to explain the indirect relationship between alexithymia and eating symptoms, such as ‘perfectionism’, that is more represented in AN than in BN patients [45].

As with other investigations in this field, our study is not exempt from limitations. We were aware that the cross-sectional design of our study responded to the requirement of immediacy of results but did not allow an accurate evaluation of the cause-effect relationships. We decided to utilize self-report questionnaires administered online, mainly for their speed of execution, but we were aware of the risk of recall bias from subjects who agreed to participate. As already stated, no measures were taken to verify the actual satisfaction of inclusion criteria. In this case, it cannot be excluded that there were subjects in the group examined who did not meet the stated inclusion criteria. Finally, the absence of a comparison group with a diagnosis of an eating disorder made the comparison between the scores of interoceptive confusion and alexithymic traits between a clinical and a non-clinical sample impossible. However, it is well-known that patients with AN or BN could be characterized by interoceptive confusion/inaccuracy and by alexithymic traits; as already noticed, few data are available on general population samples.

## 5. Conclusions

Our study, even if with the above-mentioned limitations, highlighted the role of interoceptive confusion as the dimension linking the occurrence of alexithymic traits and eating spectrum manifestations, in a non-clinical sample. We believe that interoceptive confusion could be a marker for the development of subsequent difficulties in identifying/describing feelings, that, in turn could characterize subjects at risk of developing eating signs and symptoms. This finding might have several practical and theoretical implications, raising question about the possibility of an intervention strategy on interoceptive confusion and alexithymic traits in a longitudinal instead of a more limited cross-sectional perspective, with the aim to prevent the onset of a full-blown eating disorder in general population samples. We believe that the proposed assessment should be considered for such subjects who might have a benefit from specific information and psycho-educational supports, centered on the promotion of interoceptive accuracy and awareness.

## Figures and Tables

**Table 1:** Sociodemographic Information for the overall sample and by gender.

	Overall Sample *n* = 161	Males *n* = 47	Females *n* = 114
Age ^1^	Mean/SD	Mean/SD	Mean/SD
	23.2 ± 2.4	23.7 ± 2.4	22.9 ± 2.3
Relationship Status	*n*/%	*n*/%	*n*/%
Single	148 (91.9)	42 (89.4)	106 (93.0)
With a Partner	13 (8.1)	5 (10.6)	8 (7.0)
Working activity	*n*/%	*n*/%	*n*/%
Unemployed	7 (4.3)	4 (8.5)	3 (2.6)
Student	124 (77.0)	25 (53.2)	99 (86.8)
Farmer/Worker	7 (4.3)	7 (14.9)	-
Employee	13 (8.1)	7 (14.9)	6 (5.3)
Freelance	6 (3.7)	3 (6.4)	3 (2.6)
Retailer	2 (1.2)	1 (2.1)	1 (0.9)
Research Assistant	2 (1.2)	-	2 (1.8)
Education	*n*/%	*n*/%	*n*/%
Middle school	6 (3.7)	5 (10.6)	1 (0.9)
High school	78 (48.4)	27 (57.4)	51 (44.7)
Degree	74 (46.0)	14 (29.8)	60 (52.6)
Residency	3 (1.9)	1 (2.1)	2 (1.8)
Area of residence	*n*/%	*n*/%	*n*/%
Rural	31 (19.3)	10 (21.3)	21 (18.4)
Sub-urban	54 (33.5)	17 (36.2)	37 (32.5)
Urban	76 (47.2)	20 (42.6)	56 (49.1)

^1^ Test U Mann-Whitney for independent sample: *p* = 0.018.

**Table 2:** Scores of the administered scales in the overall sample and by gender.

	Overall Sample *n* = 161	Males *n* = 47	Females *n* = 114	*p* ^1^
EAT 26	Mean/SD	Mean/SD	Mean/SD	
Total Score	12.2 ± 10.2	11.2 ± 8.0	12.6 ± 11.1	0.003
Dieting	5.9 ± 6.3	5.2 ± 5.7	6.1 ± 6.6	0.007
Bulimia/Foodpreoccupation	4.4 ± 2.6	4.2 ± 2.2	4.5 ± 2.7	0.019
Oral Control	1.8 ± 2.8	1.6 ± 2.5	1.9 ± 3.0	0.042
ICQ Total Score	50.9 ± 8.3	52.9 ± 7.9	50.1 ± 8.4	0.110
IAS Total Score	82.8 ± 11.9	84.0 ± 9.5	82.3 ± 12.7	0.438
TAS-20				
Total Score	58.5 ± 9.7	56.9 ± 9.8	59.2 ± 9.6	0.002
Identifying feelings	18.0 ± 6.3	16.5 ± 5.9	18.6 ± 6.4	0.0001
DescribingFeelings	14.8 ± 3.3	14.7 ± 3.3	14.8 ± 3.3	0.535
Externally oriented Thinking	25.7 ± 3.4	25.6 ± 4.1	25.7 ± 3.1	0.455
EAT Threshold Score	*n*/%	*n*/%	*n*/%	χ^2^
<20	134 (83.2)	42 (89.4)	92 (80.7)	
≥20	27 (16.8)	5 (10.6)	22 (19.3)	0.181
TAS-20 Threshold Score	*n*/%	*n*/%	*n*/%	χ^2^
≤61	103 (64.0)	34 (72.3)	69 (60.5)	
>61	58 (36.0)	13 (27.7)	45 (39.5)	0.156

^1^ ANCOVA corrected for ‘age’.

**Table 3:** ICQ, IAS, TAS-20 in subjects with EAT-26 score < 20 vs. EAT-26 score ≥ 20.

	EAT < 20 (*n* = 134) Mean/SD	EAT ≥ 20 (*n* = 27) Mean/SD	*p* ^1^
ICQ Total Score	50.2 ± 6.8	54.4 ± 13.2	0.011
IAS Total Score	83.2 ± 10.9	80.7 ± 15.9	0.520
TAS-20			
Total Score	58.1 ± 9.2	60.8 ± 11.9	0.006
Identifying feelings	17.3 ± 5.8	21.5 ± 7.6	0.0001
Describing Feelings	14.8 ± 3.2	14.4 ± 3.8	0.606
Externally-oriented Thinking	25.9 ± 3.3	24.8 ± 3.7	0.206

^1^ ANCOVA corrected for ‘age’ and ‘gender’.

**Table 4:** ICQ, IAS, and EAT-26 scores in in subjects with TAS-20 score > 61 vs. TAS-20 score ≤ 61.

	TAS-20 > 61 (*n* = 103) Mean/SD	TAS-20 ≤ 61 (*n* = 58) Mean/SD	*p* ^1^
ICQ Total Score	48.7 ± 7.5	54.7 ± 8.4	0.0001
IAS Total Score	85.5 ± 11.1	79.7 ± 12.7	0.078
EAT			
Total Score	11.4 ± 11.0	13.6 ± 8.7	0.008
Dieting	5.3 ± 6.6	6.9 ± 5.5	0.013
Bulimia/Food Preoccupation	4.2 ± 2.6	4.9 ± 2.6	0.031
Oral control	1.8 ± 3.1	1.8 ± 2.3	0.086

^1^ ANCOVA corrected for ‘age’ and ‘gender’.

**Table 5:** (**a**) Correlations Analyses between ICQ, IAS, TAS-20, EAT-26 total scores and domains in the overall sample (*n* = 161). (**b**) Correlations Analyses between ICQ, IAS, TAS-20, EAT-26 total scores and domains in males’ sample (*n* = 47). (**c**) Correlations Analyses between ICQ, IAS, TAS-20, EAT-26 total scores and domains in females’ sample (*n* = 114).

	IAS Total ^a^ Score	TAS-20 Total ^a^ Score	TAS-20 Identifying ^a^ Feelings	TAS-20 Describing ^a^ Feelings	TAS-20 EOT ^a^	EAT-26 Total Score ^b^	EAT-26 Dieting ^b^	EAT-26 Bulimia/Food P. ^b^	EAT-26 Oral Control ^b^
(**a**)									
ICQ Total Score ^a^	−0.055	0.383 **	0.439 **	0.122	0.150	0.302 **	0.247 **	0.232 **	0.117
IAS Total Score	1	−0.359 **	−0.460 **	−0.230 **	−0.061	−0.170 *	−0.144	−0.143	−0.087
TAS-20 Total Score	-	1	0.847 **	0.720 **	0.557 **	0.269 **	0.226 **	0.182 *	0.131
TAS-20 Identifying Feelings	-	-	1	0.409 **	0.145	0.360 **	0.301 **	0.264 **	0.195 *
TAS-20 Describing Feelings	-	-	-	1	0.312 **	0.113	0.080	0.085	0.015
TAS-EOT	-	-	-	-	1	−0.019	−0.09	−0.012	−0.067
EAT-26 Total Score	-	-	-	-	-	1	0.900 **	0.722 **	0.576 **
EAT-26 Dieting	-	-	-	-	-	-	1	0.688 **	0.300 **
EAT-26 Bulimia/Food P.	-	-	-	-	-	-	-	1	0.219 **
(**b**)									
ICQ Total Score	0.072	0.297 *	0.393 **	0.063	0.091	0.409 **	0.265	0.393 **	0.099
IAS Total Score	1	−0.435 **	−0.333 *	−0.465 **	−0.117	0.066	0.184	−0.140	−0.124
TAS-20 Total Score	-	1	0.807 **	0.626 **	0.709 **	0.164	0.075	0.108	0.205
TAS-20 Identifying Feelings	-	-	1	0.244	0.288 *	0.333 *	0.170	0.301 *	0.256
TAS-20 Describing Feelings	-	-	-	1	0.327 *	−0.039	−0.106	−0.029	−0.041
TAS-EOT	-	-	-	-	1	−0.087	−0.040	−0.069	0.024
EAT-26 Total Score	-	-	-	-	-	1	0.847 **	0.579 **	0.371 *
EAT-26 Dieting	-	-	-	-	-	-	1	0.481 **	−0.026
EAT-26 Bulimia/Food P.	-	-	-	-	-	-	-	1	0.045
(**c**)									
ICQ Total Score	−0.106	0.451 **	0.503 **	0.149	0.189 *	0.255 **	0.265	0.393 **	0.099
IAS Total Score	1	−0.334 **	−0.493 **	−0.161	0.164	−0.244 **	0.184	−0.140	−0.124
TAS-20 Total Score	-	1	0.861 **	0.765 **	0.483 **	0.310 **	0.075	0.108	0.205
TAS-20 Identifying Feelings	-	-	1	0.475 **	0.078	0.373 **	0.170	0.301 *	0.256
TAS-20 Describing Feelings	-	-	-	1	0.307 **	0.161	−0.106	−0.029	−0.041
TAS-EOT	-	-	-	-	1	0.009	−0.040	−0.069	0.024
EAT-26 Total Score	-	-	-	-	-	1	0.925 **	0.767 **	0.646 **
EAT-26 Dieting	-	-	-	-	-	-	1	0.740 **	0.433 **
EAT-26 Bulimia/Food P.	-	-	-	-	-	-	-	1	0.283 **

** *p* < 0.01 (two-tailed); * *p* < 0.05; ^a^ = Pearson correlation; ^b^ = Rho Spearman correlation.

**Table 6:** Binary Logistic Regression Analysis.

	B	E.S.	Wald	df	Sig.	Exp(B)	95% CI Per EXP(B)	
							Inf.	Sup.
ICQ total score	0.073	0.029	6.191	1	0.013	1.075	1.016	1.139
IAS total score	−0.017	0.020	0.723	1	0.395	0.983	0.946	1.022
TAS-20 total score	−0.017	0.027	0.392	1	0.531	0.984	0.934	1.036
Age	−0.210	0.107	3.815	1	0.051	0.811	0.657	1.001
Gender	0.764	0.564	1.838	1	0.175	2.147	0.711	6.478

## Data Availability

Data are contained within the article.
